# Case Report: Paroxysmal hyperhidrosis as an initial symptom in a patient with anti-LGI1 encephalitis

**DOI:** 10.3389/fimmu.2022.986853

**Published:** 2022-09-23

**Authors:** Tingting Qiao, Lanlan Chen, Li Jiang, Hua Wei, Xin Chen, Xiaobo Li, Yingzhu Chen, Yao Xu

**Affiliations:** ^1^ Department of Neurology, Northern Jiangsu People’s Hospital, Medical College of Yangzhou University, Yangzhou, China; ^2^ Department of Rheumatology, Northern Jiangsu People’s Hospital, Medical College of Yangzhou University, Yangzhou, China

**Keywords:** case report, anti-LGI1 encephalitis, autoimmune encephalitis, hyperhidrosis, autoimmune epilepsy

## Abstract

Anti-leucine-rich glioma-inactivated 1 (LGI1) encephalitis is the second most common cause of autoimmune encephalitis and is characterized by cognitive impairment, psychiatric disorders, and faciobrachial dystonic seizures. In recent decades, literature reports have expanded the phenotypic spectrum associated with the LGI1 autoantibody. The present report describes the case of a 58-year-old man who presented with repetitive unilateral hyperhidrosis of the body and arm as an initial symptom and gradually developed psychiatric symptoms, involuntary movements of the face and arms, and progressive cognitive decline. Anti-LGI1 antibodies were positive in both the serum and cerebrospinal fluid at approximately 2 months after symptom onset, and the patient was, therefore, diagnosed with anti-LGI1 encephalitis. His symptoms, namely hyperhidrosis and involuntary movements, were not relieved by antiepileptic drug treatment, but responded favorably to high-dose steroid therapy and intravenous immunoglobulin. We interpreted the repetitive unilateral hyperhidrosis as possible epilepsy. Based on this case, unilateral hyperhidrosis of the body and arm as a rare neurological presentation can be added to the phenotypic spectrum of anti-LGI1 encephalitis, and early recognition of this manifestation might support timely diagnosis and treatment.

## Introduction

Anti-leucine-rich glioma-inactivated 1 (LGI1) encephalitis is now recognized as the second most common type of autoimmune encephalitis (AE), second to anti-N-methyl-d-aspartate receptor encephalitis (1, 2). The clinical features of anti-LGI1 encephalitis, namely cognitive impairment, psychiatric disorders, faciobrachial dystonic seizures (FBDS), and refractory hyponatremia, have been well-documented in the literature since LGI1 was first identified as the true antigen for AE in 2010 (3, 4). The number of confirmed cases of anti-LGI1 encephalitis has been increasing annually in recent years, and the phenotypic spectrum of neurological presentations has been expanding accordingly (5, 6). It is important to note that the diverse clinical manifestations of anti-LGI1 encephalitis hamper its early diagnosis and treatment.

Some anti-LGI1 encephalitis patients have prodromal symptoms, such as fever, headache, dizziness, fatigue, or drowsiness (6). Subacute onset of cognitive dysfunction and psychiatric symptoms has been demonstrated in most cases of anti-LGI1 encephalitis (7, 8). Seizures occur in more than 85% of patients who suffer from anti-LGI-1 encephalitis and can be the initial, primary, or in rare cases the sole manifestation of the disease (9, 10). FBDS as a subtype of seizures was deemed to be a characteristic symptom of anti-LGI1 encephalitis (7, 11). Other symptoms, such as sleep disturbance, autonomic symptoms, motor symptoms, and hyponatremia, also are well-documented (11, 12). LGI1 encephalitis is responsive to immunotherapy treatment and is related to a low mortality rate and incidence of clinical relapses (13).

Herein, we present the case of a 58-year-old man who experienced the initial symptom of repetitive unilateral hyperhidrosis and was diagnosed with anti-LGI1 encephalitis.

## Case description

**Figure 1:**
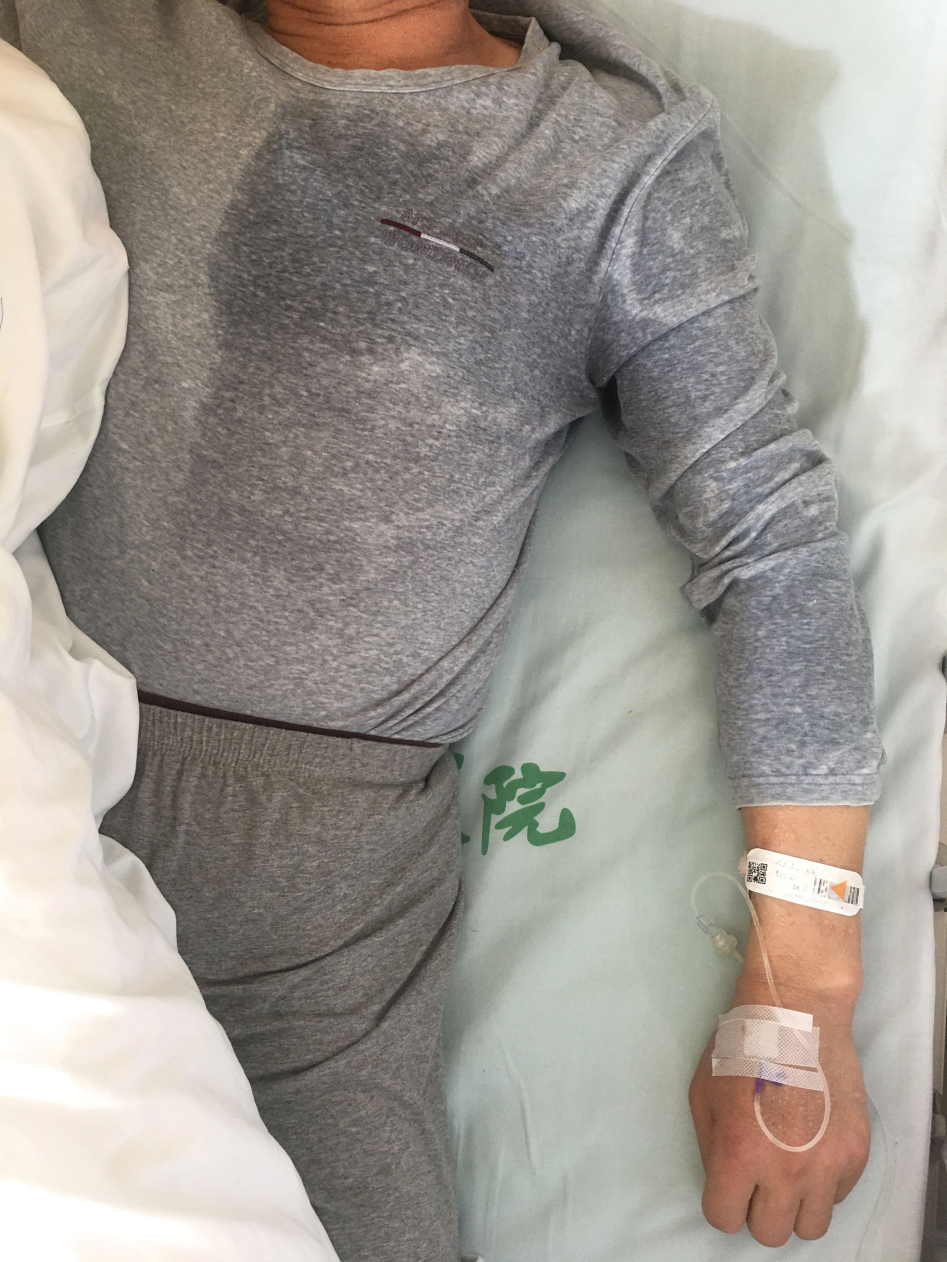
Severe hyperhidrosis of the left body and arm suddenly occurred several times every day mainly at night. The patient’s left body and arm were suddenly covered in large beads of sweat. To the contrary, his face, neck, right body, and other limbs remained dry and sweat-free. After immunotherapy, the frequency of hyperhidrosis decreased markedly with such events occurring a total of two times in the 6 months after discharge.

A 58-year-old man with no notable medical history complained of repetitive sweating from the left side of his body and left arm. He would suddenly wake during the night and find that the clothes on the left side of his body and left arm were completely soaked with sweat, whereas the naked parts of the left side of his body and arm were covered with large beads of sweat; however, the right side of his body and other limbs were dry and sweat-free (**Figure 1**; **Supplementary Figure 1**). He experienced normal sweating on the dry side both before and after these episodes. The excessive sweating occurred two to four times every day, mainly at night, and often woke the patient. After approximately 15 days, he gradually developed hallucinations, incoherent speech, and attack behavior. Another 3 days later, he was diagnosed with schizophrenia and prescribed some antipsychotic drugs. However, all symptoms continued to progress, and he became very drowsy. His wife found that the sweating episodes suddenly appeared within 2 minutes, and the frequency increased to four to six times per day. He was then hospitalized in a psychiatric hospital at 30 days after the first clinic visit.

On examination, doctors noticed that, in addition to the dominant psychiatric features, sudden jerky movements involving the face and arms were observed, which lasted approximately seconds and occurred dozens of times each day. Cranial magnetic resonance imaging (MRI) revealed cortical atrophy without other abnormalities. The patient’s serum sodium level was 127 mmol/L (normal range, 137–145 mmol/L) based on laboratory testing. The results from other serological tests, namely for thyroid hormone, autoantibodies, and tumor markers, were negative. Cerebrospinal fluid (CSF) analysis demonstrated normal protein and glucose levels without pleocytosis. An autoimmune cell-based encephalitis panel performed *via* a cell-based assay returned a positive result for anti-LGI1 antibodies in both the serum (1:32) and CSF (1:3.2). The results were negative for all of the other antibodies (anti-Neuronal NMDR, -AMPAR1, -AMPAR2, -GABAb, -CASPR2, -DPPX, -IgLON5, -GAD65, -mGluR5) in both serum and CSF.

Based on these findings, the patient was diagnosed with anti-LGI1 encephalitis and transferred to our hospital. Physical examination on admission showed somnolence and involuntary movements of the face and arms. The patient had no other autonomic symptoms, such as constipation, piloerection, tachypnea, or tachycardia upon examination of the autonomic system. Because of the extraordinarily clear boundaries of the hyperhidrosis, we did not perform a sweat test. The patient began initial treatment with high-dose methylprednisolone (500 mg daily for 5 consecutive days) and intravenous immunoglobulin (0.4 g/kg for 5 consecutive days). Meanwhile, video-electroencephalograms (EEGs) were obtained over 24 hours and demonstrated generalized slow waves without other abnormal findings. Total body computed tomography screening for malignancies yielded negative results. The cervical and thoracic MRI were normal. Before admission to our hospital, he was treated with perampanel 4 mg qn for 6 days without response; however, he had a favorable response to immunotherapy. The hyperhidrosis reoccurred only four times during his stay in our hospital. Meanwhile, involuntary movements of the face and arms, regarded as FBDS, were substantially reduced and disappeared before discharge. The mental disorders also were significantly alleviated. Because of the patient’s prominent psychiatric features, cognitive testing was first conducted on the 4th day after the start of immunotherapy, and the patient scored 11/30 on the Montreal Cognitive Assessment (MoCA) and 15/30 on the Mini-Mental State Examination (MMSE). Two weeks after the first cognitive testing, his MoCA and MMSE scores improved to 14/30 and 21/30, respectively. His serum sodium level normalized 10 days after treatment. At 24 days after the initial testing, the patient’s serum remained positive for anti-LGI1 antibody (1:32), whereas the CSF was negative for anti-LGI1 antibody. After discharge, he continued treatment with oral prednisone and mycophenolate mofetil. After 6 months, his MoCA and MMSE scores were 16/30 and 23/30. During the 6 months of follow-up, hyperhidrosis occurred only twice in the first month after discharge (**Supplementary Table 1**).

## Discussion and conclusions

This report describes a patient diagnosed with anti-LGI1 encephalitis. The clinical manifestations in this case included severe unilateral hyperhidrosis, psychiatric symptoms, FBDS, and rapidly progressive cognitive decline, and the patient’s serum and CSF were positive for anti-LGI1 antibodies. Additionally, the satisfactory response to treatment and favorable prognosis in this case further support the diagnosis of anti-LGI1 encephalitis.

Increasing numbers of anti-LGI1 encephalitis patients have been described. Prominent clinical presentations described for anti-LGI1 encephalitis include seizures, cognitive impairment, and psychiatric symptoms. These clinical symptoms are consistent with the antigenic localization and current functional understanding of LGI1. LGI1 is primarily expressed in the hippocampus and the temporal cortex and serves as a component of the voltage-gated potassium channel complex by acting as the ligand for two epilepsy-related proteins: -a disintegrin and metalloproteinase 22 (ADAM22) and ADAM23 (14).

The subacute onset of seizures is common in patients with anti-LGI1 encephalitis. Seizures can occur separately, without any other distinct encephalitis symptoms, which brings about diagnostic difficulties and treatment delay (11). FBDSs are considered pathognomonic for anti-LGI1 encephalitis but sometimes go unrecognized by patients and physicians (7, 8). Whether FBDS should be considered as a seizure type or nonepileptic seizure is widely debated. Recently, some researchers detected generalized EEG electrodecremental events, and others found typical focal contralateral frontal waves, both preceding the onset of muscle artifacts, indicating that FBDS were atypical seizures (15).

Other infrequent seizure semiologies also have been reported, including impaired awareness and motor, gelastic, aura, and focal autonomic seizures (11). However, those episodes often occurred for many months before correct recognition. Some researchers suggest the denomination of FBDS-plus to include and raise awareness of other uncommon epileptic semiologies (16). One patient of LGI1-IgG seropositivity experienced memory disturbance and suffered from frequent chest pain of a squeezing and dull nature typically lasting 10–30 seconds, and the chest pain was finally confirmed as a symptom of partial seizures by video-EEG (17). Another anti-LGI1 encephalitis patient was reported to have behavioral disturbance, cognitive impairment, and recurrent goosebumps traveling up his spine that lasted seconds and occurred multiple times a day, and the goosebumps were considered as possible autonomic auras (18). In another case, a young woman experienced only frequent limb piloerection, lasting from a few seconds to 2 minutes and occurring several to dozens of times throughout the day. Those events were finally identified as pilomotor seizures, and the patient was diagnosed with anti-LGI1 encephalitis (19). Paroxysmal-dizziness-spells (PDS), which are a newly found syndromic manifestation, are reported in 14% of anti-LGI1 encephalitis patients. These spells are considered to be partial seizures or aura phenomena that escape detection in surface EEG recordings (5).

The repetitive unilateral hyperhidrosis of the body and arm observed in our patient is an unusual manifestation, and to our knowledge, this is the only case documented to date. This abnormal pattern of sweating recurred several times per day for almost 50 days and was not resolved by treatment with antiepileptic drugs, but showed a definite response to immunotherapy. The gratifying response to immunotherapy provided a strong clue as to the immune-mediated characteristic of the hyperhidrosis. Immunotherapy is important in the treatment of anti-LGI1 encephalitis and has contributed to a significant reduction in the frequency of FBDS, which often seem unresponsive to antiepileptic drugs (20). The excessive hyperhidrosis in the present case was considered as a possible rare seizure semiology, somewhat similar to goosebumps in the one anti-LGI1 encephalitis patient mentioned above. Unilateral hyperhydrosis is sometimes seen in patients with spinal injuries, and this type of hyperhidrosis is often persistent. In our patient, the burst features of hyperhidrosis events were extremely different from those after spinal injuries. Furthermore, the spinal MRI of our patient showed no abnormalities. Therefore, the hyperhidrosis in our patient was considered a symptom of encephalitis.

Sweating abnormalities documented previously in anti-LGI1 encephalitis patients mainly include the loss of sweating in the body and limbs, which was considered an autonomic symptom (21, 22). A study by investigators at the Mayo Clinic reported that, among patients who were positive for anti-LGI1 and CASPR2 antibodies, about 25% had autonomic symptoms, of which orthostatic hypotension and reduced sweating were the most common symptoms (5). Additionally, one third of the LGI1-IgG-seropositive patients had pain with or without sweating abnormality (5). Previous LGI1 encephalitis cases also described hyperhidrosis in 13.6% of patients and attributed it to autonomic dysfunction but without many details (23). Because no positive sign was found during examination of the autonomic nervous system (such as piloerection, tachypnea, or tachycardia), the unilateral hyperhidrosis with paroxysmal and repetitive features was interpreted as possible epilepsy. However, it was not confirmed by surface EEG, perhaps due to the absence of the episode during the video-EEG process after immunotherapy.80112

The phenotypic spectrum of neurological presentations continues to expand as more patients with LGI1 autoantibodies are identified. Notably, the unilateral hyperhidrosis associated with LGI1 antibodies in our patient is a rare manifestation. This report can help raise awareness of the possibility of anti-LGI1 encephalitis in patients who experience this symptom to avoid delaying diagnosis.

## Patient perspective

The patient and his family were very glad for the fast improvement in his condition after he received immunotherapy. Upon learning that his condition is quite rare, the patient consented to share his case.

## Data availability statement

The original contributions presented in the study are included in the article/**Supplementary Material**. Further inquiries can be directed to the corresponding author.

## Ethics statement

The study involving human participant was reviewed and approved by Ethics Committee of Northern Jiangsu People's Hospital. The patient provided his written informed consent to participate in this study. Written informed consent was obtained from the individual for the publication of any potentially identifiable images or data included in this article.

## Author contributions

TQ, LC, YX, and YC treated the patient. XL, YC and HW collected and interpreted the autoimmune encephalitis panel. LJ was responsible for follow up. TQ and LC wrote the manuscript and are responsible for the writing. All authors approved the submitted version of the report.

## Conflict of interest

The authors declare that the research was conducted in the absence of any commercial or financial relationships that could be construed as a potential conflict of interest.

## Publisher’s note

All claims expressed in this article are solely those of the authors and do not necessarily represent those of their affiliated organizations, or those of the publisher, the editors and the reviewers. Any product that may be evaluated in this article, or claim that may be made by its manufacturer, is not guaranteed or endorsed by the publisher.

## Supplementary material

Supplementary Figure 1 Repetitive unilateral hyperhidrosis of the left body and arm happened several times every day before immunotherapy.
