# Supplementary figures and images for: Case Report: Paroxysmal hyperhidrosis as an initial symptom in a patient with anti-LGI1 encephalitis
Source: Front Immunol. 2022 Sep 23;13:986853. doi: 10.3389/fimmu.2022.986853 (PMC9537696; doi:10.3389/fimmu.2022.986853)

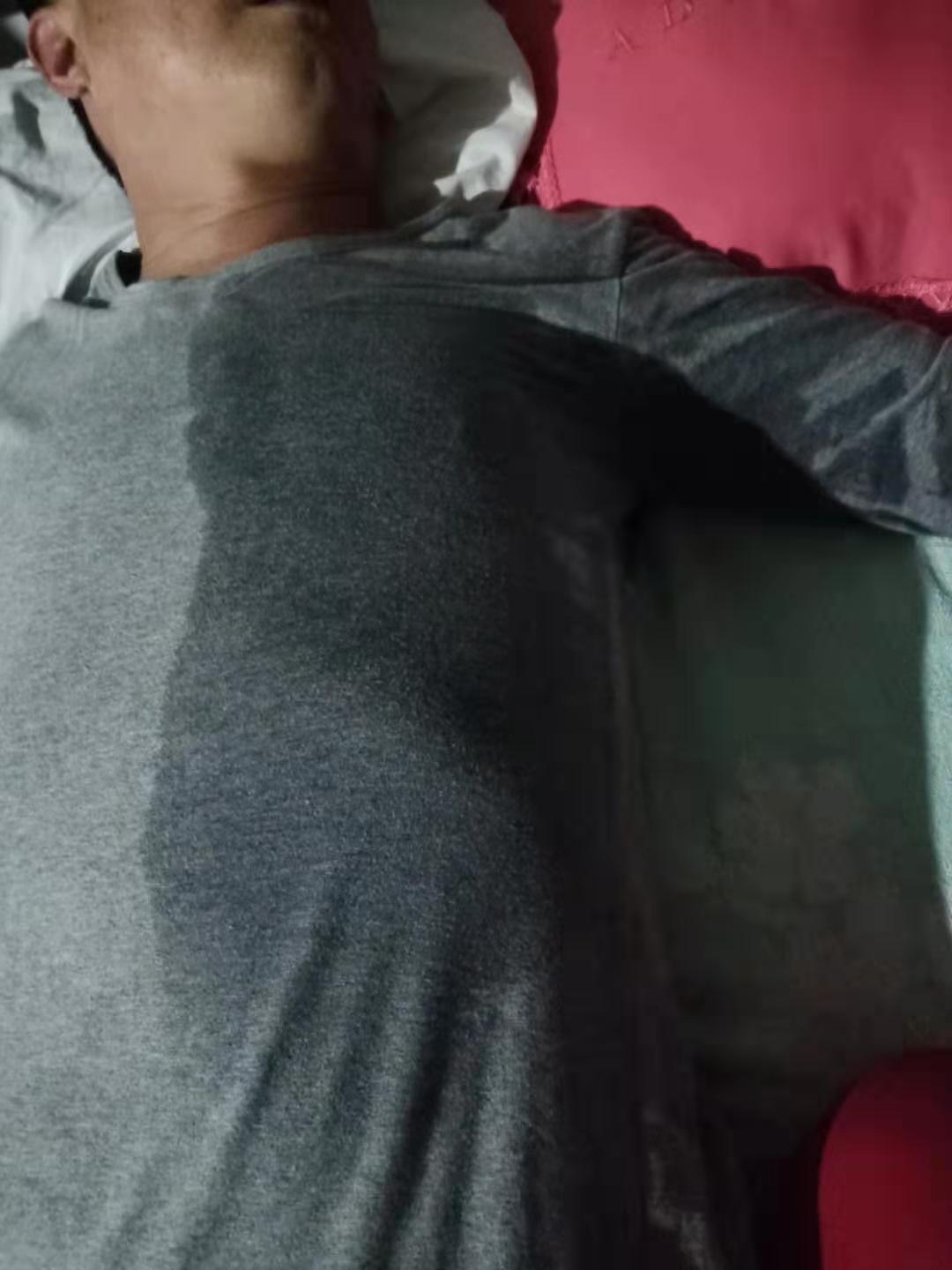

Supplement: Supplementary Figure 1 — Repetitive unilateral hyperhidrosis of the left body and arm happened several times every day before immunotherapy. [file Image_1.jpeg]
