# Supplementary material for: Case Report: Paroxysmal hyperhidrosis as an initial symptom in a patient with anti-LGI1 encephalitis
Source: Front Immunol. 2022 Sep 23;13:986853. doi: 10.3389/fimmu.2022.986853 (PMC9537696; doi:10.3389/fimmu.2022.986853)
Supplement: Supplementary file 2 [file Table_1.docx]

Supplementary Table 1| Timeline with relevant data from the patient’s course of treatment.

| Date | Symptoms, treatment and outcome |
| --- | --- |
| 2021.10.02 | Hyperhidrosis |
| 2021.10.17 | Psychiatric symptoms |
| 2021.11.18 | FBDS, cognitive impairment |
| 2021.11.23 | Anti-LGI1 antibodies were detected in serum (1:32) and CSF (1:3.2) |
| 2021.11.24 | Start of immunotherapy |
| 2021.11.30 | FBDS and psychiatric symptoms were alleviated; 11/30 on MoCA and 15/30 on MMSE |
| 2021.12.12 | Anti-LGI1 antibodies were detected in serum (1:32) and not detected in CSF; 14/30 on MoCA and 21/30 on MMSE |
| 2022.06.15 | Hyperhidrosis occurred twice in the first month after discharge and not again during the follow-up period; 16/30 on MoCA and 23/30 on MMSE |

Note: FBDS, faciobrachial dystonic seizures; anti-LGI1, anti-leucine-rich glioma-inactivated 1; CSF, Cerebrospinal fluid; MoCA, Montreal Cognitive Assessment; MMSE, Mini-Mental State Examination.
